# Utilization of a breast cancer risk assessment tool by internal medicine residents in a primary care clinic: impact of an educational program

**DOI:** 10.1186/s12885-019-5418-6

**Published:** 2019-03-14

**Authors:** Siddhartha Yadav, Sarah Hartkop, Paola Yumpo Cardenas, Rand Ladkany, Alexandra Halalau, Sandor Shoichet, Michael Maddens, Dana Zakalik

**Affiliations:** 1 Division of Medical Oncology, Mayo Clinic, 200 First Street SW, Rochester, MN 55905 USA; 2 Department of Internal Medicine, Beaumont Health, 3601 W 13 Mile Rd, Royal Oak, MI 48073 USA; 3 Nancy and James Grosfeld Cancer Genetics Center, Beaumont Cancer Institute, Beaumont Health, 3577 W 13 Mile Rd, Suite 140, Royal Oak, MI 48073 USA; 4 Oakland University William Beaumont School of Medicine, 2200 N Squirrel Rd, Rochester, MI 48309 USA

**Keywords:** Gail, BCRAT, Breast Cancer, Risk assessment, Internal medicine, Residents, Primary care physicians, Chemoprevention, Knowledge

## Abstract

**Background:**

Despite strong evidence of benefit, breast cancer risk assessment and chemoprevention are underutilized by primary care physicians. This study evaluates the impact of an educational program on knowledge and utilization of the NCI Breast Cancer Risk Assessment Tool (BCRAT) by internal medicine residents.

**Methods:**

Internal medicine residents at the primary care clinic at William Beaumont Hospital participated in an educational program on breast cancer risk assessment and chemoprevention. A questionnaire was used to assess knowledge and practice before and after participation. Electronic health records of women between the ages of 35 and 65 who were seen by participating residents for annual health exams between Dec 15, 2015 and Dec 14, 2016 were reviewed. Utilization of BCRAT by the residents was compared pre- and post-educational program.

**Results:**

A total of 43 residents participated in the study. 31 (72.1%) residents reported no prior knowledge about BCRAT. The remaining 12 (27.9%) reported limited knowledge of BCRAT, but the majority of these (*n* = 10, 83.3%) had not used it in the last six months. For each question on the pre-educational knowledge assessment, fewer than 10% of the residents responded correctly. After implementation of the educational program, there was a significant increase in the proportion of residents who answered correctly (Range: 67 to 100%, *p* < 0.001).

Electronic health records of 301 clinic patients were reviewed, 118 (39.2%) in the pre-educational program group and 183 (60.8%) in the post-educational program group. There was a higher use of BCRAT in the post-educational program group compared to the pre-intervention group (3.8% vs. 0%, *p* < 0.05). However, a majority (*n* = 294, 98.7%) of eligible patients from both groups did not undergo breast cancer risk assessment.

**Conclusions:**

Our study demonstrates that an educational intervention improved residents’ knowledge of BCRAT. Despite this improvement, a significant proportion of patients did not undergo breast cancer risk assessment. Expanding the scope and duration of this intervention and combining it with innovative use of technology to improve utilization should be the subject of future investigation.

**Electronic supplementary material:**

The online version of this article (10.1186/s12885-019-5418-6) contains supplementary material, which is available to authorized users.

## Background

One in eight women in the United States will develop invasive breast cancer in her lifetime [1]. In 2017, it was estimated that 252,710 new cases of breast cancer were diagnosed and 40,610 women died from it [2]. Although it is impossible to predict with certainty who will develop breast cancer, clinicians can identify women who may be at an increased risk and provide them with options for early detection and risk reduction. A number of validated, quantitative risk-assessment models incorporate features of a patient’s medical and family history to accurately estimate their individual risks [3]. Examples of such risk assessment models in use today include the Gail model [4], the Claus model [5] and the Tyrer-Cuzick model [6], each with its own unique strengths and limitations.

The Gail model was originally designed in 1989 [4] and then modified in 1999 [7]. The modified Gail model, also known as the National Cancer Institute (NCI)-Gail model or the Breast Cancer Risk Assessment Tool (BCRAT), is available on the NCI’s website (https://bcrisktool.cancer.gov/). The National Comprehensive Cancer Network guidelines endorse the BCRAT to identify women who may be candidates for chemoprevention with one of the currently approved agents for risk reduction. [8]. Similar guidelines issued by American Society of Clinical Oncology [9] and United States Preventative Services Task Force [10] also advocate for use of BCRAT and other breast cancer risk assessment tools to identify women who will benefit from chemoprevention. BCRAT is one of the most widely used breast cancer risk assessment tools by primary care physicians (PCPs) due to its simplicity and easy accessibility on the internet [11].

Several important medical decisions may be impacted by knowing a woman’s underlying risk of developing breast cancer [12]. For women 35 years old with a five-year projected risk of ≥1.67% by BCRAT, chemoprevention with tamoxifen, raloxifene, or exemestane should be considered [8]. Use of tamoxifen in this high-risk population decreases the risk of breast cancer by 49% [13]. For patients with a lifetime breast cancer risk of 20–25%, as defined by family history based risk assessment model, MRI is recommended as a supplement to mammography [14]. The sensitivity of MRI in this high-risk population is significantly higher, ranging from 71 to 100%, compared to 16 to 40% for mammography [14]. In addition, risk assessment and identification of women who are at a higher risk of developing breast cancer can lead to referral to a high risk breast clinic; this, in turn, has the potential to identify breast cancer at an early stage and improve outcomes [15].

Despite the availability of these breast cancer risk assessment tools and their clinical validation, they have received very little attention outside of the oncology clinic [12]. The use of breast cancer risk assessment tools by PCPs is less than 25% [16, 17]. This is concerning as women without a personal history of breast cancer are primarily managed by PCPs. For these women to benefit from breast cancer risk reduction strategies, it is important that their PCPs be aware of and able to use risk assessment models in daily clinical practice.

Our study focuses on one specific group of PCPs – internal medicine residents – since a prior study has demonstrated that resident PCPs are even less likely to use breast cancer risk assessment tools compared to their attending physicians [16]. Hence, in this study, we evaluated the utilization of BCRAT by internal medicine residents at William Beaumont Hospital, Royal Oak.

## Methods

The primary objective of this study was to evaluate the impact of a comprehensive educational program on the knowledge and utilization of BCRAT by internal medicine residents. The secondary objective was to evaluate the feasibility of such an educational program.

**Fig. 1:**
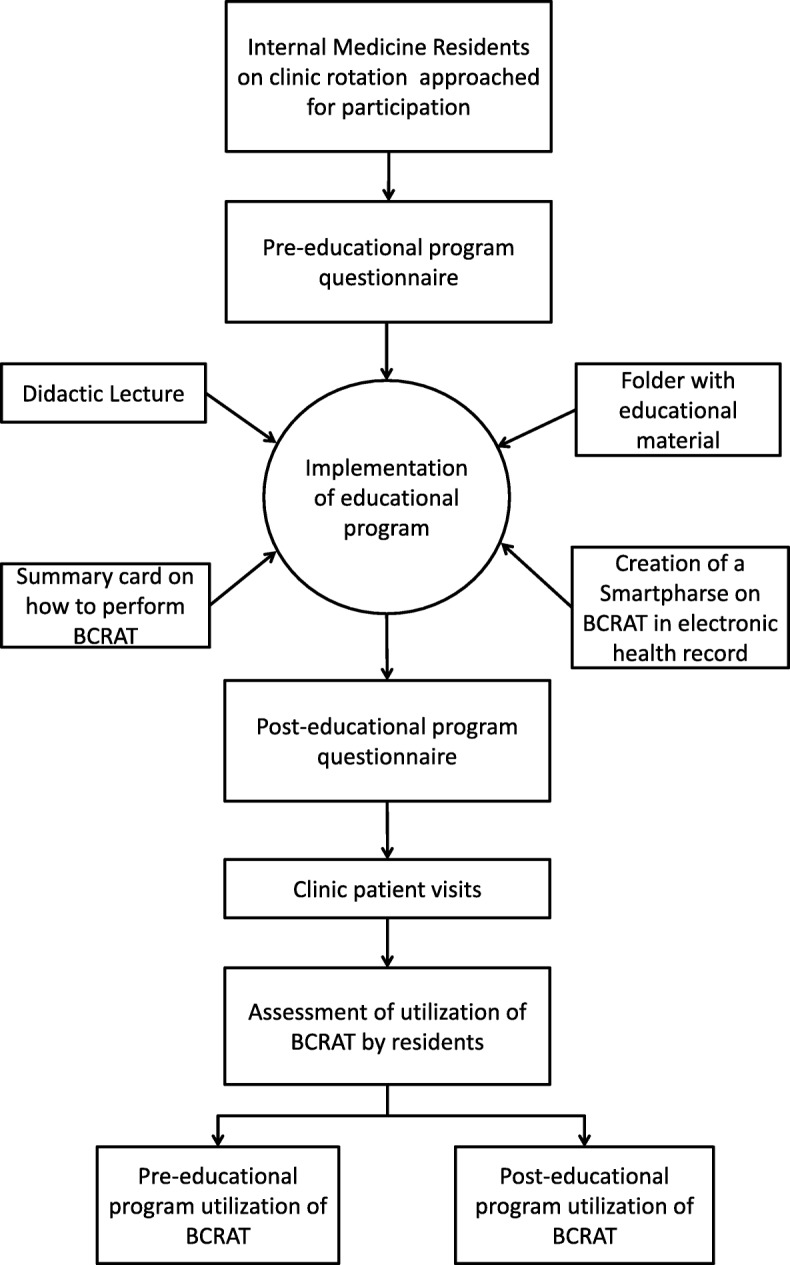
Study Design

The study was conducted over an eight-month period, from April 2016 to November 2016. Each month internal medicine residents (PGY-1 through PGY-3) assigned to an outpatient clinic rotation were offered participation. The residents took part in a thirty-minute interactive educational program at the beginning of their clinic month. The program consisted of a didactic lecture focused on breast cancer risk assessment, chemoprevention and importance of high-risk screening. In addition, the residents were provided with current literature including professional societies’ guidelines, on the topic of breast cancer risk assessment and pharmacologic prevention. A smart-phrase was created in the electronic health system (EPIC) to assist in performing BCRAT. Pre- and post-educational program questionnaire (Additional file 1) consisting of multiple choice questions was used to assess the residents’ knowledge and practice prior to the educational program, and to measure the change in knowledge (Fig. 1).

In order to evaluate the utilization of BCRAT in clinical practice, electronic health record (EHR) of women between the ages of 35 and 65 who were seen by the participating residents in the clinic for annual health exams between December 15, 2015 and December 14, 2016 were reviewed. Women with a prior history of breast cancer or a known genetic mutation were excluded. Utilization of BCRAT by the residents was assessed pre- and post-program implementation.

Data was collected in Microsoft Excel (Ver. 2007) and statistical analysis was performed using SPSS 21(IBM Corp. Released 2012. IBM SPSS Statistics for Windows, Version 21.0. Armonk, NY:IBM Corp.). Fischer’s exact test was used for categorical variables and Mann-Whitney U-Test was used for continuous variables. All tests were two sided. Statistical significance was considered at *p* < 0.05.

## Results

A total of 43 (54.4%) out of the eligible 79 residents participated in the study. A majority of these residents were graduates of a United States/Canadian medical school and were in the first year of their residency training (Table 1).

**Table 1:** Baseline Characteristics of participating residents

	*n* = 43
Level of training	
PGY – 1	22 (51.2%)
PGY – 2	9 (20.9%)
PGY – 3	12 (27.9%)
Medical School	
US/Canada	23 (53.5%)
International	16 (37.2%)
Unknown	4 (9.3%)

### Pre- and post-educational program questionnaire

All participating residents (*n* = 43) completed the pre-educational program questionnaire. When asked about their comfort level in assessing a woman’s risk for developing breast cancer, using an incremental linear scale from 0 to 10, more than half (58.1%) of the residents responded with a score of 5 or less (Median score: 5; Mode: 5). 31 (72.1%) residents stated that they had never heard of the Gail model or the NCI BCRAT before. The remaining 12 residents (27.9%) had heard of the Gail model but 10 (83.3%) out of the 12 stated they had never used it in the last six months.

In the pre-program questionnaire on knowledge about BCRAT, more than two-thirds of the residents answered each question incorrectly or did not select an answer. There was a significant increase (*p* < 0.001) in the proportion of correct answers in the post-program questionnaire, with more than two-thirds answering each of the questions correctly (Table 2).

**Table 2:** Comparison between pre- and post-educational program questionnaire survey

Multiple choice question headings	Number of residents with correct answer		
	Pre-educational program (*n* = 43)	Post-educational program (*n* = 43)	*p*-value
Q1. What is the Gail Model?	3 (7.0%)	43 (100%)	< 0.001
Q2. Who should you perform the Gail model on?	4 (9.3%)	35 (81.4%)	< 0.001
Q3. The Gail model is applicable for women over what age:	1 (2.3%)	41 (95.3%)	< 0.001
Q4. What is classified as high risk in the Gail model?	1 (2.3%)	43 (100%)	< 0.001
Q5. Who should be considered for MRI for breast cancer screening based on breast cancer risk assessment per the American Cancer Society?	3 (7.0%)	36 (83.7%)	< 0.001
Q6. Women who have an elevated 5-year risk of breast cancer should be offered	2 (4.7%)	41 (95.3%)	< 0.001
Q7. Correctly identify all variables incorporated into the Gail Model (multiple selections required to be called as correct answer)	2 (4.7%)	29 (67.4%)	< 0.001

### Utilization of BCRAT

EHR of 301 patients seen for annual physical exams by the participating residents were analyzed.118 (39.2%) patients had been seen by the residents prior to their participation in the educational program (pre-educational program group), while 183 (60.8%) were seen after the residents’ participation in the program (post-educational program group). Both groups of patients were similar in terms of their baseline demographic characteristics (Table 3).

**Table 3:** Baseline characteristics of patients

	Total (*n* = 301)	Pre-educational program patient group (*n* = 118)	Post-educational program patient group (*n* = 183)	*p*-value
Age				
Mean age at visit (years)	50.5	49.9	50.9	0.35
Median age at visit (years)	51.0	49.0	51.0	0.11
Range (years)	35–65	35–65	35–65	
Race				0.61
Caucasian	136 (45.2%)	49 (41.5%)	87 (47.5%)	
African American	121 (40.2%)	52 (44.1%)	69 (37.7%)	
Asian	1 (0.5%)	0 (0%)	1 (0.5%)	
Other	24 (8.0%)	8 (6.8%)	16 (8.7%)	
Unknown	19 (6.3%)	9 (7.6%)	10 (5.5%)	
Family History (First degree only)				0.44
0	263 (87.4%)	100 (84.7%)	163 (89.1%)	
At least one first degree relative with breast cancer	38 (12.6%)	18 (15.2%)	20 (10.9%)	
1	32 (10.6%)	15 (12.7%)	17 (9.3%)	
2	5 (1.7%)	3 (2.5%)	2 (1.1%)	
3	1 (0.3%)	0 (0.0%)	1 (0.3%)	
Menarche				
Unknown	218 (72.4%)	81 (68.6%)	137 (74.9%)	
Mean age (years)	12.4	12.5	12.4	0.91
Median age (Years)	12.0	12.0	12.0	0.84
Range	9–18	9–15	9–18	
Age at first live birth				1.00
No live birth	25 (8.3%)	10 (8.5%)	15 (8.2%)	
Unknown	236 (78.4%)	92 (78.0%)	144 (78.7%)	
Mean age (Years)	22.20	24.06	20.95	0.08
Median age (Years)	21.00	21.50	21.00	0.31
Range	15–41	16–41	15–29	
History of breast biopsy				0.84
No	270 (89.7%)	105 (89.0%)	165 (90.2%)	
Yes	31 (10.3%)	13 (11.0%)	18 (9.8%)	
1	23 (7.6%)	8 (6.8%)	15 (8.2%)	
2	7 (2.3%)	4 (3.4%)	3 (1.6%)	
3	1 (0.3%)	1 (0.8%)	0 (0.0%)	

In total, 7 (3.8%) patients in the post-educational group underwent breast cancer risk assessment. There was a modestly higher use of BCRAT in the post-educational group of patients compared to the pre-educational group (3.8% vs. 0%, *p* < 0.05). Although the sample of the residents in each sub-group was small, there was no difference in the likelihood of performing BCRAT by place of training (US/Canada vs. Other) or level of training (Post-graduate year) for the residents.

A total of 294 (97.7%) patients, 118 from the pre-educational program group and 176 from the post-educational program group, did not have a breast cancer risk assessment performed. Out of these 294 patients, 40 (13.3%) had sufficient information in the chart to calculate their Gail risk retrospectively, and 5 (12.5%) out of these 40 women had a 5-year risk of breast cancer ≥1.67%. For the remaining 254 patients, Gail risk could not be calculated from the existing information in EHR. Of these 254, 102 patients were from the pre-educational group while 152 patients were from the post-educational group, constituting 86.4 and 83.0% of their corresponding groups respectively. In more than two-thirds of the patients, information on age at menarche and age at first live birth was not available in the EHR, thus making the BCRAT calculation not possible. However, among these patients, 28 (11%) had at least one first degree relative with breast cancer.

## Discussion

Our study demonstrates that a comprehensive lecture-based educational program improved the knowledge of BCRAT in internal medicine resident PCPs. A prior study that evaluated the role of a multimodal curriculum to teach internal medicine residents evidence-based breast health demonstrated improvement in knowledge after implementation of the program, but did not evaluate the impact on clinical practice [18]. To the best of our knowledge, our study is the first one to evaluate the impact of an educational program on both knowledge and practice of internal medicine resident PCPs regarding BCRAT.

In our study, more than two-third of the residents expressed that they had no knowledge of the Gail model/BCRAT prior to implementation of our program, and among those who knew about it, the self-reported utilization was minimal. Evaluation of EHR of 118 eligible patients who were seen by these residents prior to their participation in the program revealed that none of the patients had breast cancer risk documented. Although the utilization rate of BCRAT by PCPs has been shown to be low in prior studies [16, 17, 19], the rate observed in our study is much lower. However, in contrast to our study, which looked directly into the EHR for utilization data, most of the prior studies relied on self-reported utilization rates and also included attending physicians.

The low rate of utilization of breast cancer risk assessment by resident PCPs is concerning. Whether this observation holds true in other residency programs is not known, but warrants further investigation. Our study serves as a cautionary reminder for other training programs to evaluate their curriculum and to emphasize on utilization of BCRAT in the context of teaching early detection and prevention of breast cancer. Considering the fact that residency training impacts future practice behavior [20, 21], it is imperative that residents become familiar with breast risk assessment tools so that they will carry this knowledge into their future practice. Furthermore, the resident staffed clinics often serve a patient population with a disproportionately larger share of complex medical, psychosocial and financial or insurance issues [22]. Since disparities in health care correlates with adverse outcomes in patients with breast cancer [23], it is even more important that resident PCPs are thoroughly engaged in primary prevention of breast cancer in this population.

Chemoprevention agents such as tamoxifen and raloxifene have demonstrated clear benefit in reducing risk of breast cancer in high-risk women [13, 24–26]. Despite this, they are significantly underutilized in the primary care clinic [27]. One of the proposed reasons for underutilization of chemoprevention is lack of knowledge, training or time on part of the PCPs to assess a woman’s risk of developing breast cancer [28]. In our study, 12.5% of patients for whom the Gail risk was calculated by the study investigators retrospectively were found to have an elevated five-year risk of breast cancer. These patients would potentially benefit from a balanced discussion about chemoprevention, and as such may represent missed opportunities for preventing breast cancer.

On a more positive note, our study demonstrates that internal medicine residents are willing and able to absorb knowledge on breast cancer risk assessment and prevention. However, despite this improvement in knowledge and understanding, the uptake of BCRAT increased only to a modest 3.8%. Our study did not assess for reasons behind why the residents did not assess breast cancer risk despite having adequate knowledge to do so. There are several potential reasons for the low uptake including time constraint in a busy clinic, not attributing enough clinical significance to breast cancer risk assessment over other preventative measures and not being required to perform BCRAT by attending physicians. Future studies should aim to explore some of these potential reasons. Our study also suggests that knowledge of BCRAT may not be the only barrier to implementing a successful program on breast cancer risk assessment.

Our study primarily focused on didactic lecture as the means of conveying information, but future studies should evaluate more innovative methods such as team-based or problem-based learning techniques, which may be more effective than the traditional lecture forum [29–32]. Also, encouraging use of mobile applications to calculate Gail risk may also improve utilization. It is notable that more than two-thirds of the patients did not have any information in EHR on date of menarche or first live birth, two important components of BCRAT. These clinical parameters are considered very useful in an office visit with a gynecologist but it is likely that not enough significance is attributed to these clinical parameters in an internal medicine clinic, and hence were likely not collected prior to or during the patient visit. Embedding BCRAT into EHR or allowing patients to input their information related to BCRAT in EHR might help residents more easily identify patients at an increased risk. While clinic attendings were by design not part of our intervention, they play an important role in resident education and supervision, and can further reinforce and encourage the residents to use BCRAT.

There are several limitations of our study including the small number of residents from a single institution and limited scope of our intervention. We did not evaluate the long-term impact of our educational program nor did we specifically look at reasons for low uptake. We did not evaluate or educate the residents on how to effectively communicate the results of BCRAT to patients, which is another limitation. Future studies should build upon our findings and include ongoing education to reinforce the important concepts of breast cancer risk assessment and prevention.

## Conclusions

Our study demonstrates that an educational program geared towards resident PCPs improves knowledge and utilization of BCRAT. Despite this improvement, there is a need to expand the scope and duration of this intervention in order to optimize the use of BCRAT in a busy clinic. Combining educational programs with other teaching modalities and innovative use of technology may further improve the utilization of BCRAT and therefore, should be investigated in future studies.

## Additional file

Additional file 1: Pre- and Post-educational Questionnaire. Description: List of questions used in pre- and post-educational questionnaire for the resident physicians. (DOCX 20 kb)

## Abbreviations

BCRAT: Breast Cancer Risk Assessment Tool
EHR: Electronic Health Record
NCI: National Cancer Institute
PCPs: Primary Care Physicians
