# Supplementary material for: Utilization of a breast cancer risk assessment tool by internal medicine residents in a primary care clinic: impact of an educational program
Source: BMC Cancer. 2019 Mar 14;19:228. doi: 10.1186/s12885-019-5418-6 (PMC6416938; doi:10.1186/s12885-019-5418-6)
Supplement: Supplementary file 1 — Pre- and Post-educational Questionnaire. Description: List of questions used in pre- and post-educational questionnaire for the resident physicians. (DOCX 20 kb) [file 12885_2019_5418_MOESM1_ESM.docx]

**Additional file 1:**

**Pre-educational program questionnaire**

PGY_______

Mother’s date of birth (mm/dd/yyyy): ____________

Med school (Check one): US/Canada International

1. On a scale of 0 to 10 (0 being not comfortable at all, and 10 being very comfortable), how comfortable are you assessing a woman’s risk for breast cancer?

______

1. Have you ever heard about the Gail Model also known as the NCI Breast cancer risk assessment tool?

___ Yes

___ No

1. How often have you used the Gail model or any other similar tools in your clinical practice in the last 6 months?
   1. Never
   2. Rarely (1-3 times in the last 6 months)
   3. Sometimes (4-10 times in the last 6 months)
   4. Often (>10 times in the last 6 months)
2. If you have never used gail model in your clinic practice in the last 6 months (option ‘a’ of question 1), please state why you have not done so (Encircle all that are applicable)
   1. I was not sure what it was
   2. I did not know where to find it
   3. It takes too much of my time
   4. I did not think it was relevant to my patients
   5. Other_______
3. How often do you take a family history of breast cancer in your female patients? Please encircle one.
   1. Always
   2. Sometimes
   3. Rarely
   4. Never
4. What is the Gail model?
   1. I have heard about it but I do not know what it is
   2. It is a tool that predicts the risk of having a genetic mutation such as BRCA 1 or 2 in young women
   3. It predicts the risk of recurrence in women with personal history of breast cancer
   4. It predicts the risk of breast cancer in women without cancer
   5. None of the above
5. Who should you perform the Gail model on?
   1. Women with a history of breast cancer
   2. Women with a BRCA1 or 2 mutation
   3. Women without a history of breast cancer or genetic mutation
   4. Women who refuse any form of breast cancer screening
   5. Women who have at least one first degree relative with breast cancer
6. The Gail model is applicable for women:
   1. ≥ 18 years of age
   2. ≥ 40 years of age
   3. ≥ 35 years of age
   4. ≥ 50 years of age
   5. ≥ 45 years of age
7. What is classified as high risk in the Gail model
   1. 5-year risk greater than 5%
   2. 5-year risk greater than 1.67%
   3. 5-year risk greater than 3.37%
   4. 5-year risk greater than 10%
   5. 5-year risk greater than 5.57%
8. Who should be considered for MRI for breast cancer screening based on gail model per the American Cancer Society?
   1. Women with life time risk of breast cancer >10%
   2. Women with life time risk of breast cancer >30%
   3. Women with life time risk of breast cancer >20%
   4. Women with life time risk of breast cancer >5%
   5. Women with life time risk of breast cancer >40%
9. Women who have an elevated 5-year risk of breast cancer should be offered:
   1. Mammograms every 3-months
   2. Mammograms every 6-months
   3. Genetic testing for BRCA 1 or BRCA 2 mutation
   4. Chemoprevention with Tamoxifen
   5. Bilateral mastectomy
10. Where or who should you refer your patients at Beaumont health who are at high risk based on The Gail Model?
    1. Cancer genetics center for genetic testing
    2. High risk breast clinic
    3. To a breast surgeon for mastectomy
    4. To an oncologist
    5. To a radiation oncologist
11. Which of the following variables are incorporated into the Gail Model? Please encircle all that are applicable:
    1. Age of the patient
    2. Age at first menstrual period
    3. Age at first live birth of a child
    4. Number of first degree relatives with breast cancer
    5. History of breast biopsy
    6. Race/ethnicity
    7. History of mammograms
    8. None of the above

**Post-educational program questionnaire**

PGY_______

Mother’s date of birth (mm/dd/yyyy): ____________

1. What is the Gail model?
   1. I have heard about it but I do not know what it is
   2. It is a tool that predicts the risk of having a genetic mutation such as BRCA 1 or 2 in young women
   3. It predicts the risk of recurrence in women with personal history of breast cancer
   4. It predicts the risk of breast cancer in women without cancer
   5. None of the above
2. Who should you perform the Gail model on?
   1. Women with a history of breast cancer
   2. Women with a BRCA1 or 2 mutation
   3. Women without a history of breast cancer or genetic mutation
   4. Women who refuse any form of breast cancer screening
   5. Women who have at least one first degree relative with breast cancer
3. The Gail model is applicable for women:
   1. ≥ 18 years of age
   2. ≥ 40 years of age
   3. ≥ 35 years of age
   4. ≥ 50 years of age
   5. ≥ 45 years of age
4. What is classified as high risk in the Gail model
   1. 5-year risk greater than 5%
   2. 5-year risk greater than 1.67%
   3. 5-year risk greater than 3.37%
   4. 5-year risk greater than 10%
   5. 5-year risk greater than 5.57%
5. Who should be considered for MRI for breast cancer screening based on gail model per the American Cancer Society?
   1. Women with life time risk of breast cancer >10%
   2. Women with life time risk of breast cancer >30%
   3. Women with life time risk of breast cancer >20%
   4. Women with life time risk of breast cancer >5%
   5. Women with life time risk of breast cancer >40%
6. Women who have an elevated 5-year risk of breast cancer should be offered:
   1. Mammograms every 3-months
   2. Mammograms every 6-months
   3. Genetic testing for BRCA 1 or BRCA 2 mutation
   4. Chemoprevention with Tamoxifen
   5. Bilateral mastectomy
7. Where or who should you refer your patients at Beaumont health who are at high risk based on The Gail Model?
   1. Cancer genetics center for genetic testing
   2. High risk breast clinic
   3. To a breast surgeon for mastectomy
   4. To an oncologist
   5. To a radiation oncologist
8. Which of the following variables are incorporated into the Gail Model? Please encircle all that are applicable:
   1. Age of the patient
   2. Age at first menstrual period
   3. Age at first live birth of a child
   4. Number of first degree relatives with breast cancer
   5. History of breast biopsy
   6. Race/ethnicity
   7. History of mammograms
   8. None of the above
